# Supplementary figures and images for: Association of Bidirectional Network Cores in the Brain with Perceptual Awareness and Cognition
Source: J Neurosci. 2025 Feb 27;45(17):e0802242025. doi: 10.1523/JNEUROSCI.0802-24.2025 (PMC12019110; doi:10.1523/JNEUROSCI.0802-24.2025)

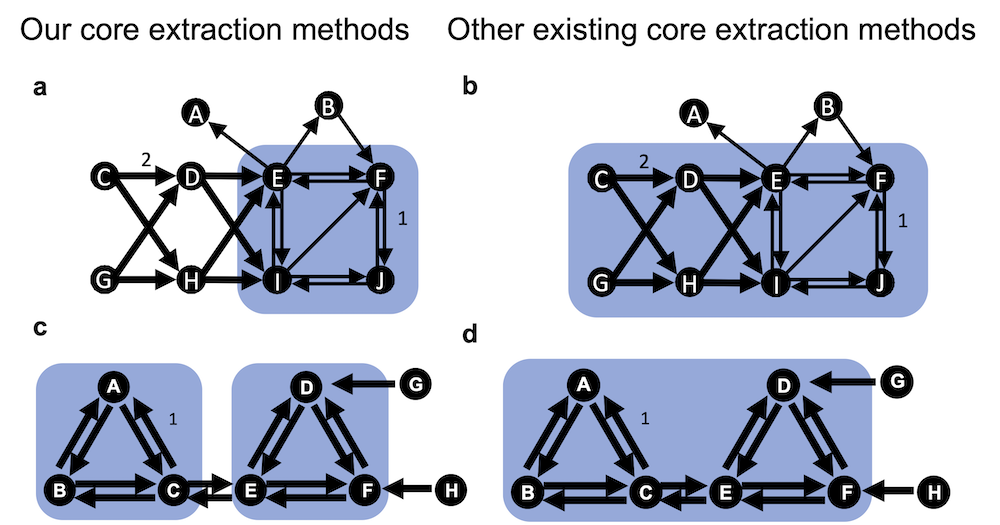

Supplement: Figure 1-1 — Examples of the differences between our core extraction method and other core extraction methods. a, b, Example of a graph W illustrating the difference in bidirectionality of the extracted cores. This is the same as Figure. 1d. In this case, our method extracts only the bidirectionally connected subnetwork (a node set {EFIJ}) as the most central core (a), whereas s-core decomposition extracts a subnetwork that include unidirectionally connected nodes (e.g., nodes C, D, G, H) as the most central core (b, a node set {CDEFGHIJ}). In the case of s-core decomposition, it was applied to the symmetrized undirected network (W + WT)/2. c, d, Example illustrating the difference in globality of core extraction. In this network, two modules (a sub-graph containing nodes A, B, C and that containing nodes C, D, E) are connected by bidirectional edges. The weight of all edges is set to 1. In this case, other existing core extraction methods (such as s-core/k-core decomposition) extract a single, nearly entire network as the most central core (d), whereas our method extracts two sub-networks as the most central cores (c). Download Figure 1-1, TIF file. [file jneuro-45-e0802242025-s004.tif]

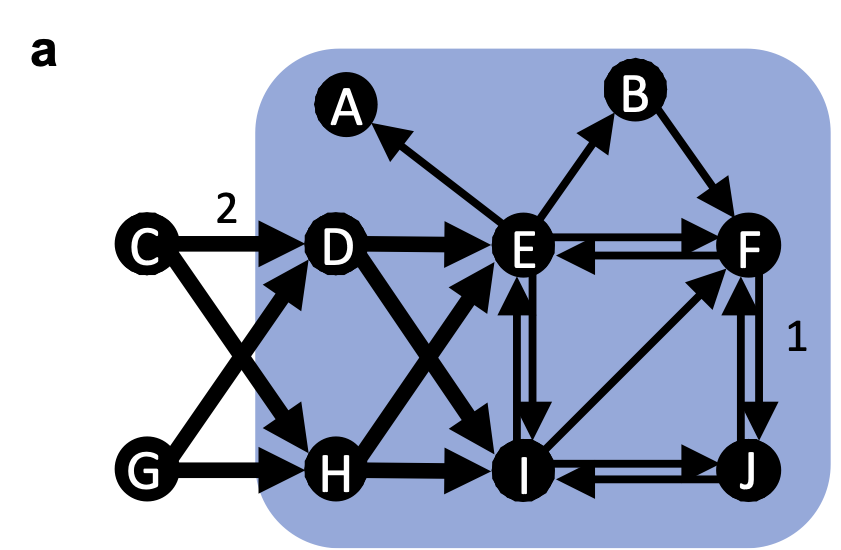

Supplement: Figure 1-2 — An example of extracted cores by the functional rich club (Deco et al., 2021). a, The network is the same as Figure. 1d. The functional rich club method extracts a subnetwork that include unidirectionally connected nodes (e.g., node A,D,H) as the most central core (b, a node set {ABDEFHIJ}). Here, GFRIC(k) is defined as GFRIC(k)=∑i∈k∑j∈kWij+∑i∈k∑jWij−∑i∑j∈kWij (where k is a subset of regions k = {i1, i2, …, il}), and the core was identified as the largest subnetwork among those whose GFRIC(k) fall within the top 5% of subnetworks of the same size. Download Figure 1-2, TIF file. [file jneuro-45-e0802242025-s005.tif]

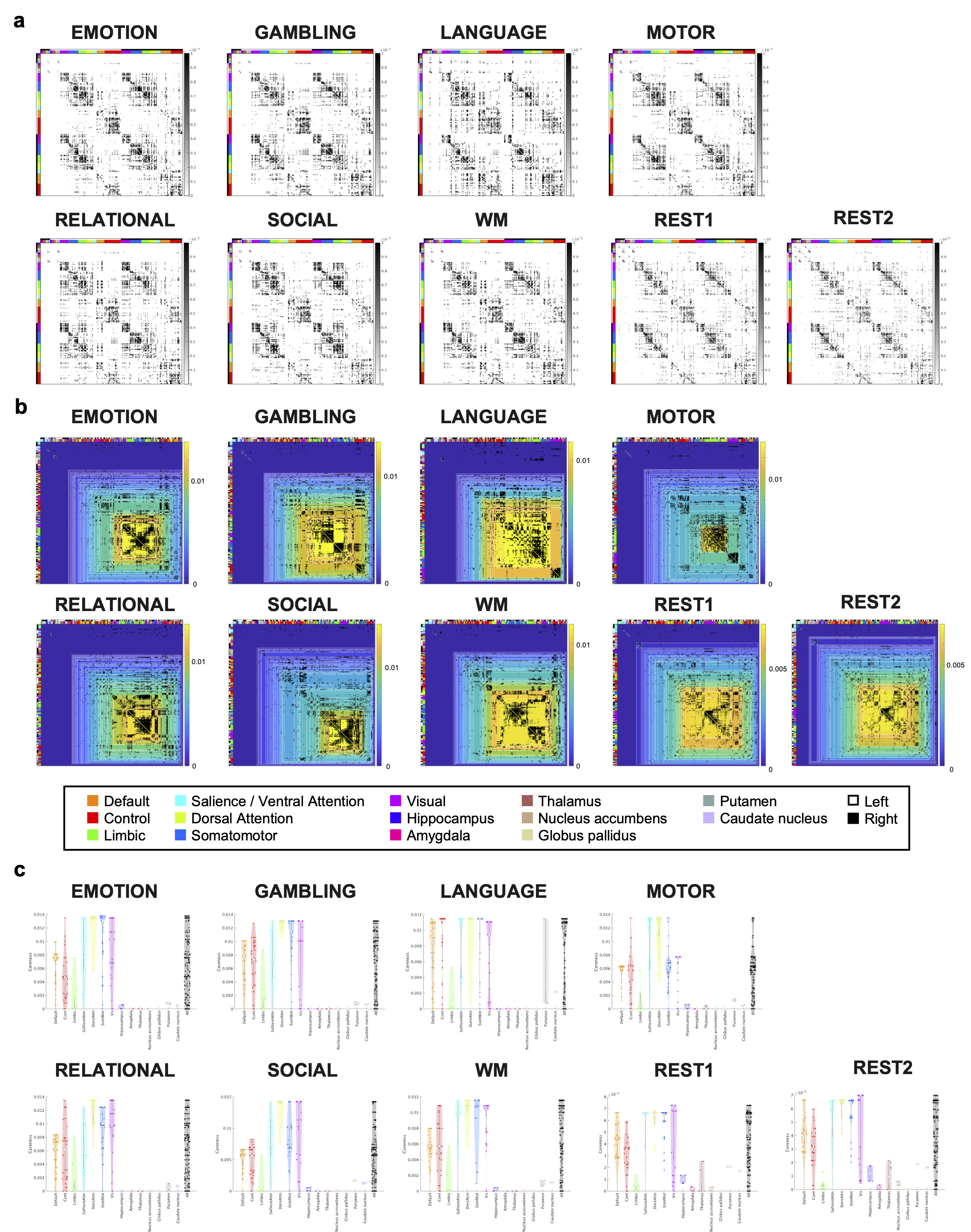

Supplement: Figure 2-1 — Results of the bidirectional cores extracted for each task. a, Estimated directed graphs at rest and during seven tasks. The grayscale of each matrix element represents the weight of the edge in the estimated directed network. The white and black at the left and top of the matrix indicate the left and right hemispheres, respectively, whereas the color bar represents the divisions of the Yeo-7 network atlas and major divisions of subcortical areas. b, Hierarchical structure of the cores with bidirectional connections at rest and during seven tasks. The directed network is arranged according to the hierarchical structure of the complexes, with white lines separating each hierarchical level. c, Violin plots showing the distribution of coreness at rest and during seven different tasks, categorized by the Yeo-7 network atlas for cortical regions and major divisions for subcortical areas. a, b, c show, from left to right, the results for emotion, gambling, language, motor, relational, social, working memory, rest1 and rest2, respectively. Download Figure 2-1, TIF file. [file jneuro-45-e0802242025-s006.tif]

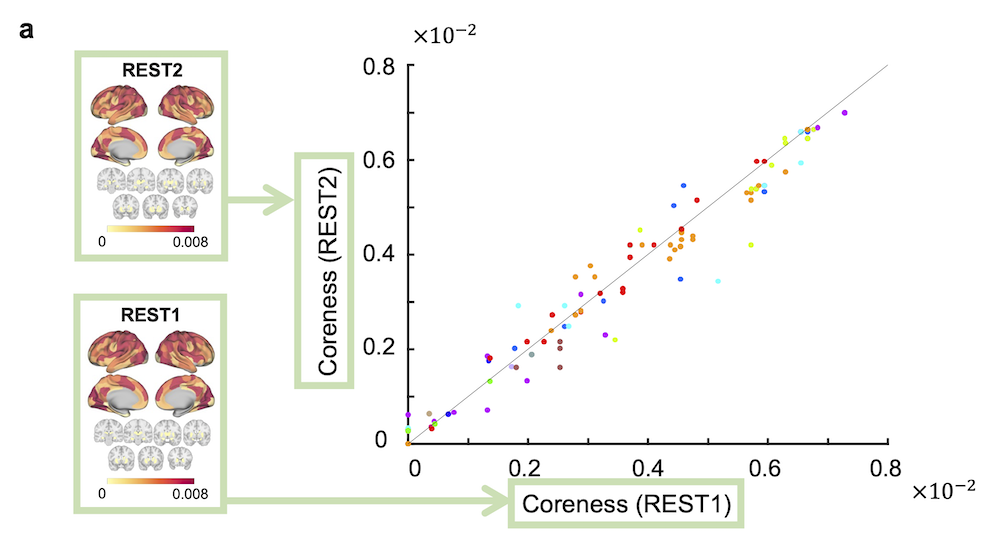

Supplement: Figure 2-2 — The similarity of coreness between Rest 1 and Rest 2. a, (Left:) Coreness at Rest 1 (top) and Rest 2 (bottom) in the cerebral cortex and the subcortex shown in seven coronal slices. (Right:) A scatterplot of coreness at Rest 1 and Rest 2. The solid line represents the identity line (y = x). Download Figure 2-2, TIF file. [file jneuro-45-e0802242025-s007.tif]

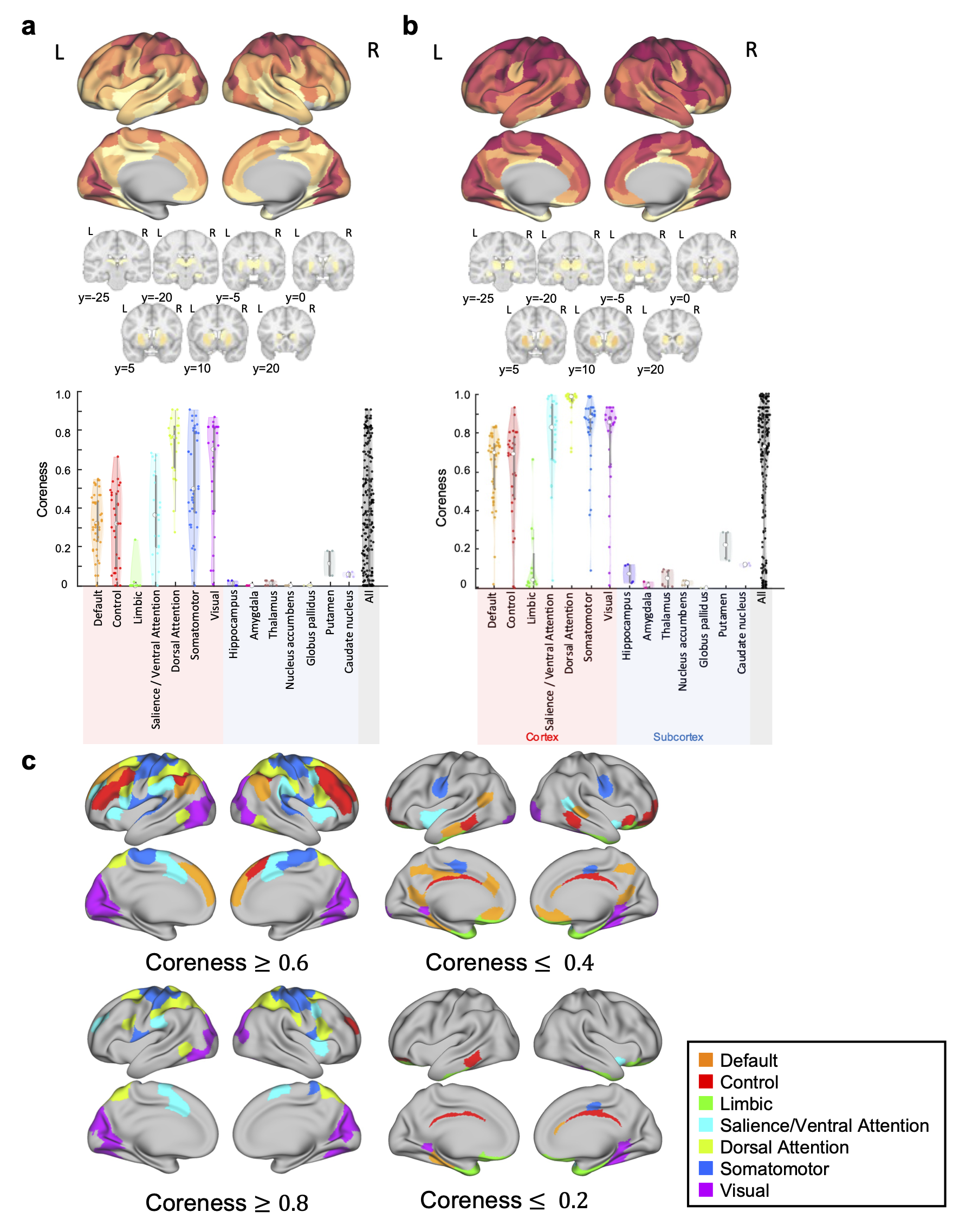

Supplement: Figure 2-3 — The robustness of trends in the extracted bidirectional cores when graph density or thresholding value of coreness is varied. a, b, Coreness at 5% (a) and 20% (b) graph density in the cerebral cortex (top) and the subcortex shown in seven coronal slices (middle), along with violin plots of coreness (bottom). c, ROIs with coreness greater than 0.6 or 0.8, or less than 0.4 or 0.3, are colored according to the Yeo-7 network atlas. Download Figure 2-3, TIF file. [file jneuro-45-e0802242025-s008.tif]

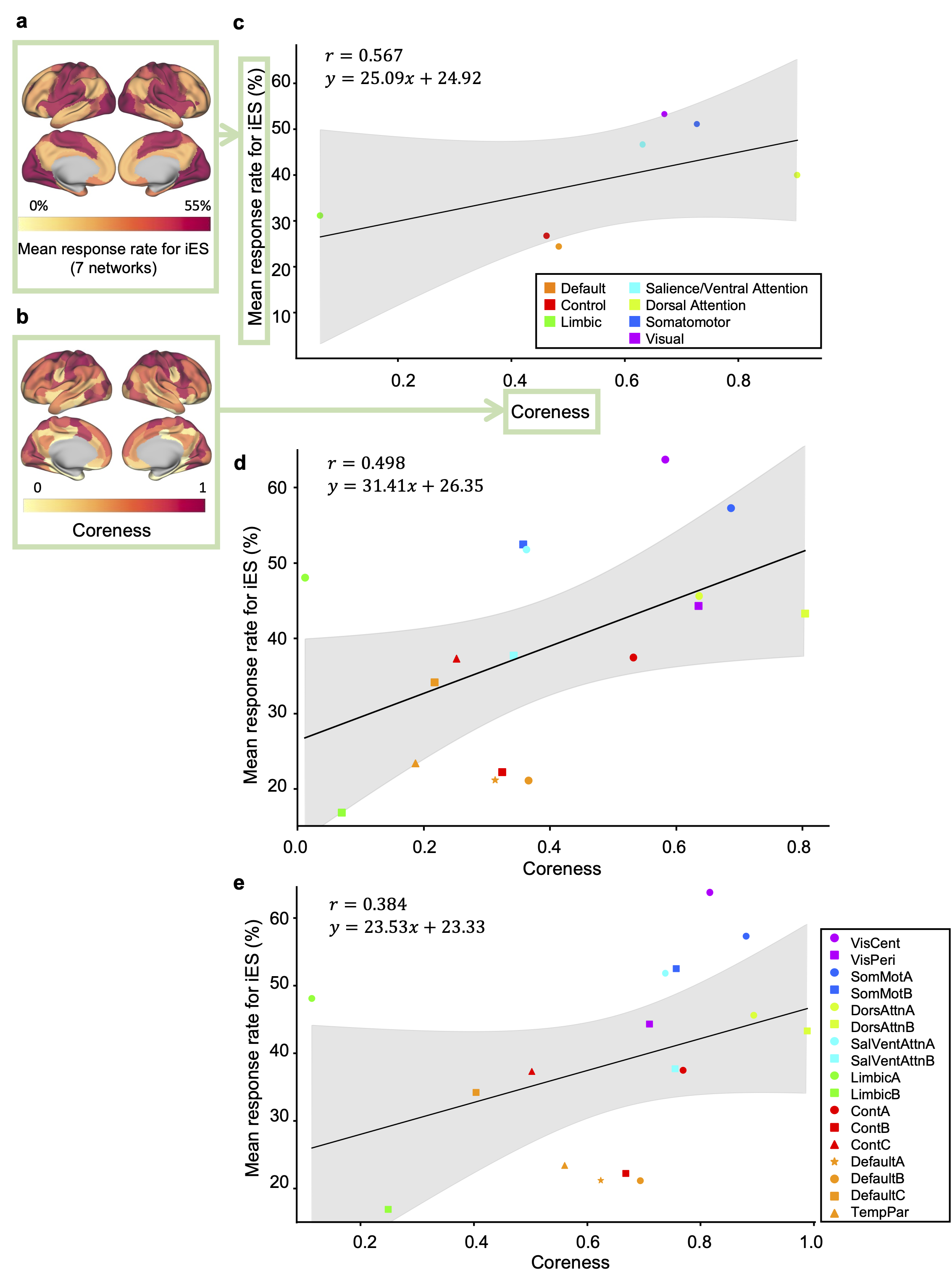

Supplement: Figure 3-1 — The robustness of the trend of positive correlation between coreness and MRR when Yeo-7 network atlas is used and graph density is varied. a, b, A cortical surface rendering of the MRR for iES (7 network) (a), and that of coreness (b). c, A scatter plot showing the average coreness for each division of the Yeo-7 network atlas (horizontal axis) and the MRR (vertical axis). The solid line represents the regression line. Each point is color-coded according to the Yeo-7 network atlas. d, e, Scatter plots showing the average coreness for each division of the Yeo-17 network atlas (horizontal axis) and the MRR (vertical axis) at 5% graph density (d) and 20% graph density (e). Download Figure 3-1, TIF file. [file jneuro-45-e0802242025-s009.tif]

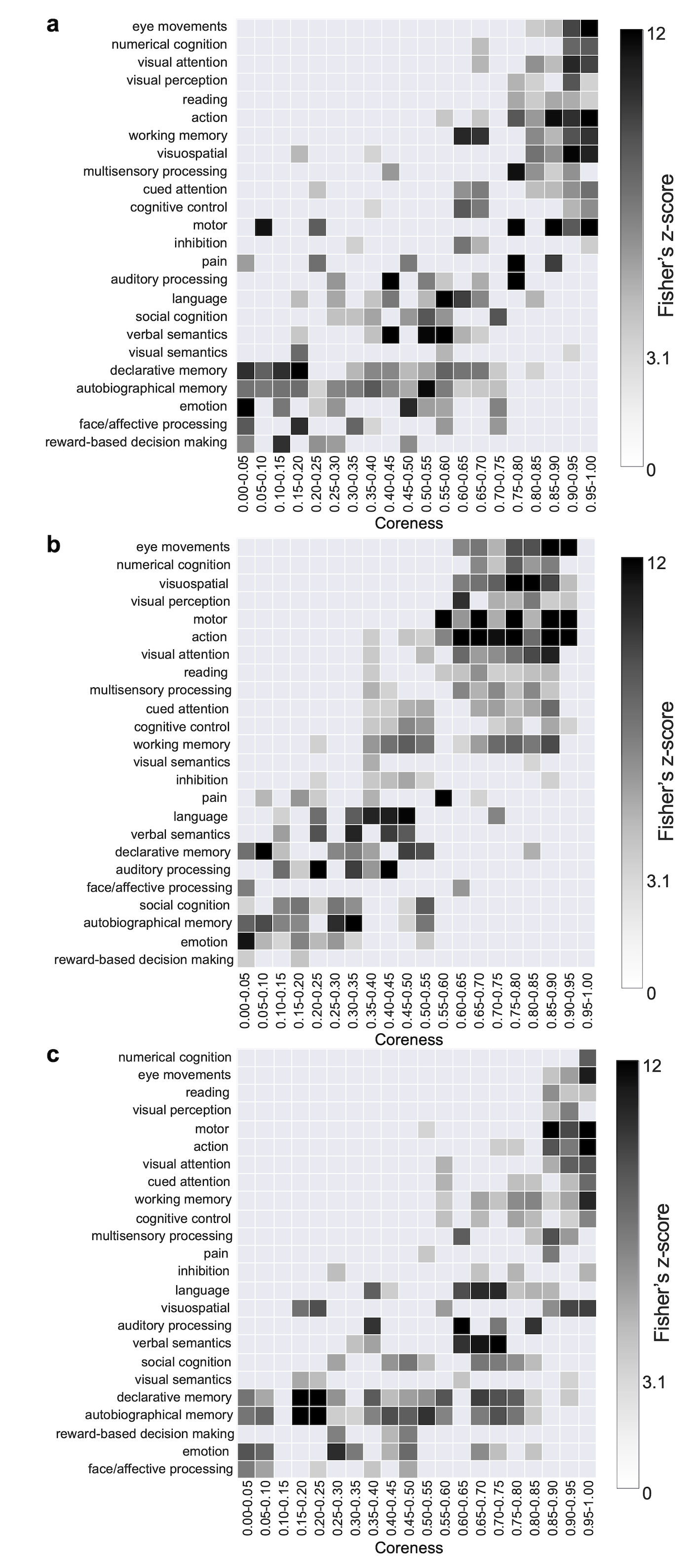

Supplement: Figure 4-1 — The robustness of the observed relationship found in the Term-based meta-analysis of the core structure using NeuroSynth when subcortical regions were included and graph density was varied. a, b, c, A term-based meta-analysis using NeuroSynth applied to coreness when subcortical regions were included (a), and when graph density was 5% (b) and 20% (c). Download Figure 4-1, TIF file. [file jneuro-45-e0802242025-s010.tif]

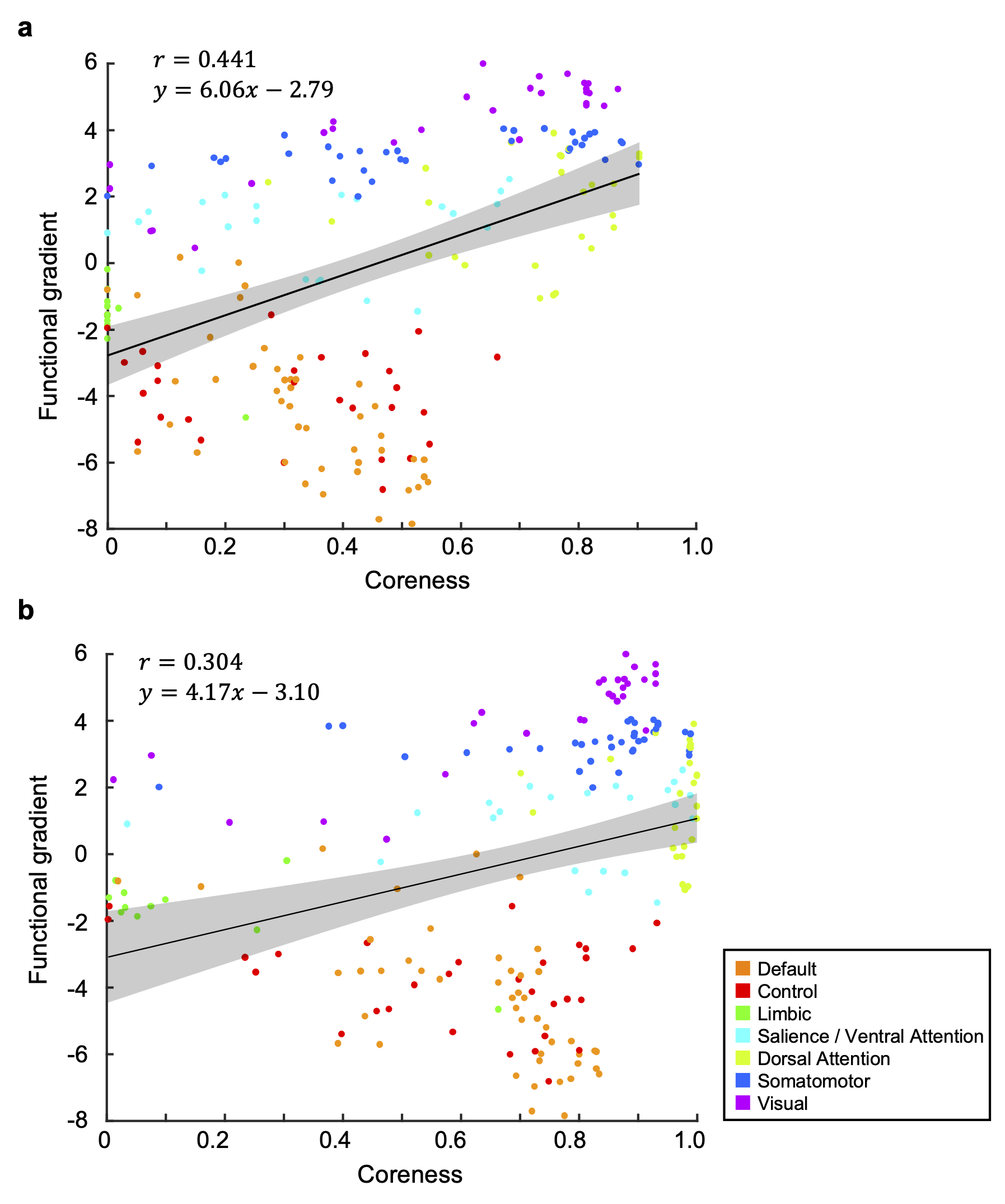

Supplement: Figure 5-1 — The stability of the positive correlation between coreness and the functional connectivity gradient when graph density was varied. a, b, Scatterplots of coreness and the functional connectivity gradient when graph density was 5% (a) and 20% (b). Download Figure 5-1, TIF file. [file jneuro-45-e0802242025-s011.tif]

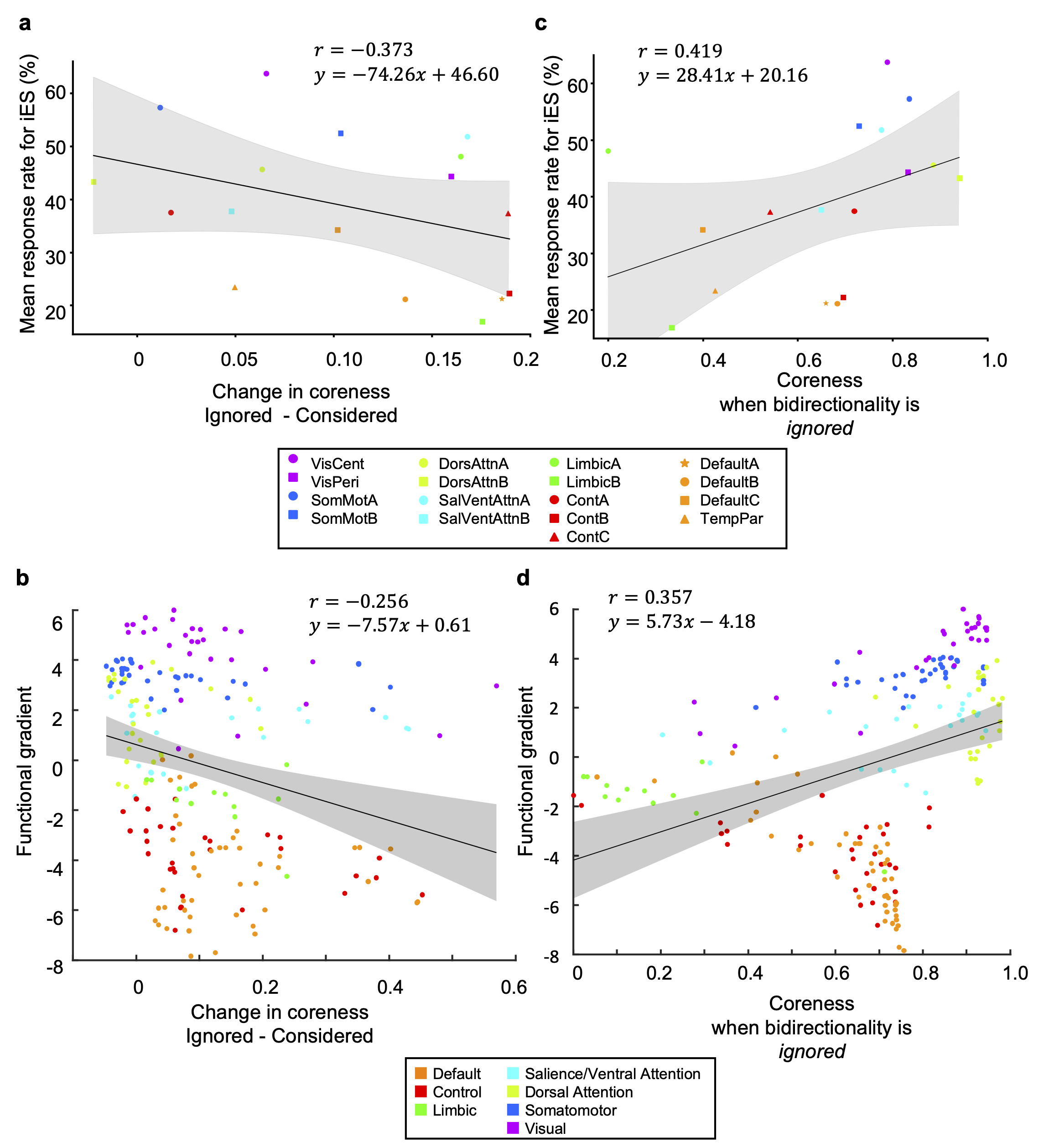

Supplement: Figure 6-1 — Comparison of changes in coreness when ignoring bidirectionality and coreness when ignoring bidirectionality with the MRR for iES and the functional connectivity gradient. a, There is a negative correlation between the change in coreness and the MRR for iES (r = −0.373). b, There is a negative correlation between the change in coreness and the functional connectivity gradient (r = −0.256). The solid lines represent the regression lines. c, Compared to when bidirectionality is considered, the correlation between coreness and the MRR is weaker when bidirectionality is ignored (r = 0.419). d, Compared to when bidirectionality is considered, the correlation between coreness and the functional connectivity gradient is weaker when bidirectionality is ignored (r = 0.357). The solid lines represent the regression lines. Download Figure 6-1, TIF file. [file jneuro-45-e0802242025-s012.tif]

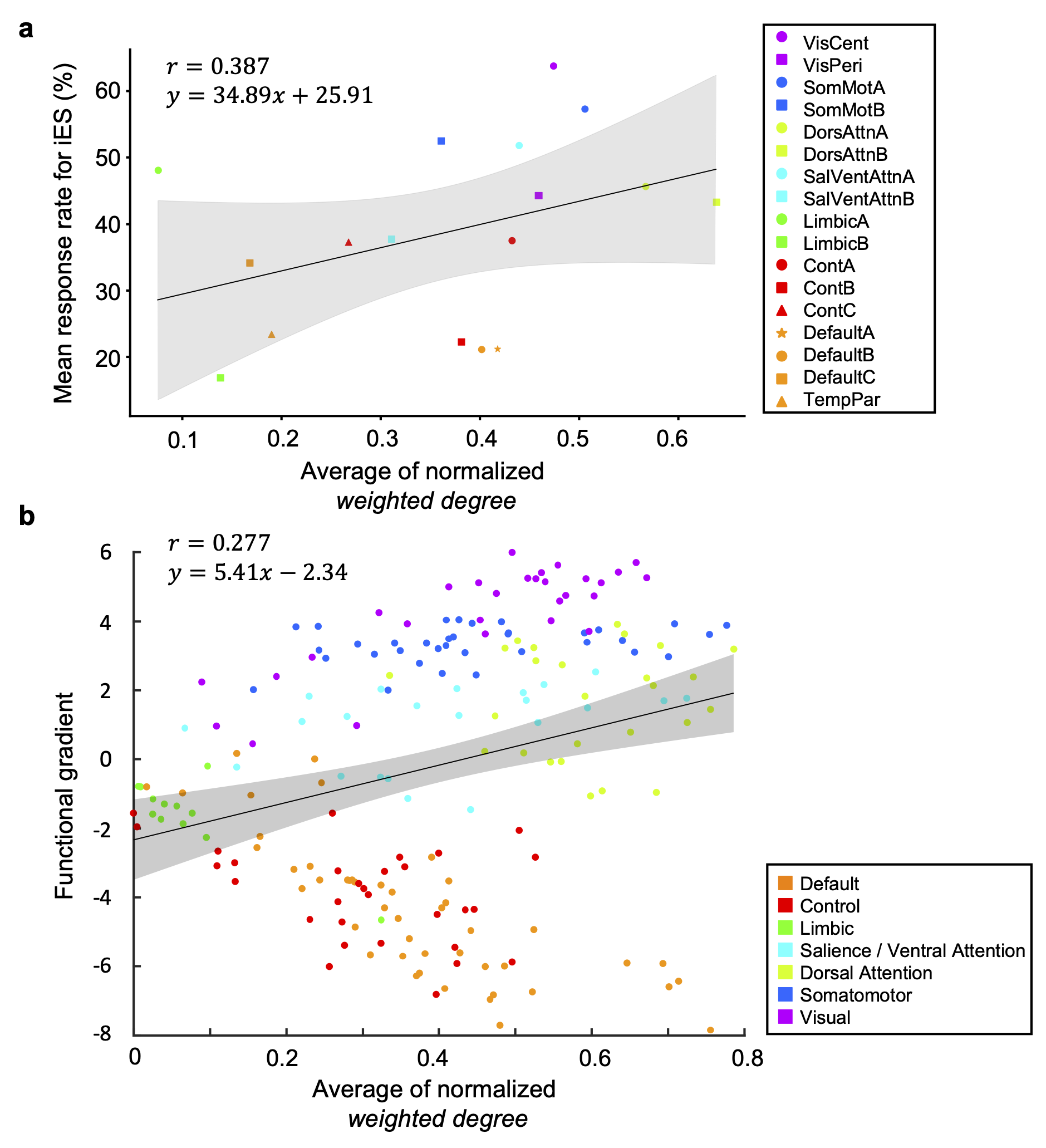

Supplement: Figure 7-1 — Comparison of weighted degree with the MRR for iES and the functional connectivity gradient. a, The correlation between the weighted degree and the MRR (r = 0.387) is weaker than that between the coreness when bidirectionality is considered and the MRR. b, The correlation between the weighted degree and the functional connectivity gradient (r = 0.277) is weaker than that between coreness when considering bidirectionality and the functional connectivity gradient. The solid lines represent the regression lines. Download Figure 7-1, TIF file. [file jneuro-45-e0802242025-s013.tif]
